# Supplementary material for: The long non-coding RNA MIR31HG regulates the senescence associated secretory phenotype
Source: Nat Commun. 2021 Apr 28;12:2459. doi: 10.1038/s41467-021-22746-4 (PMC8080841; doi:10.1038/s41467-021-22746-4)

Supplementary Figure 1

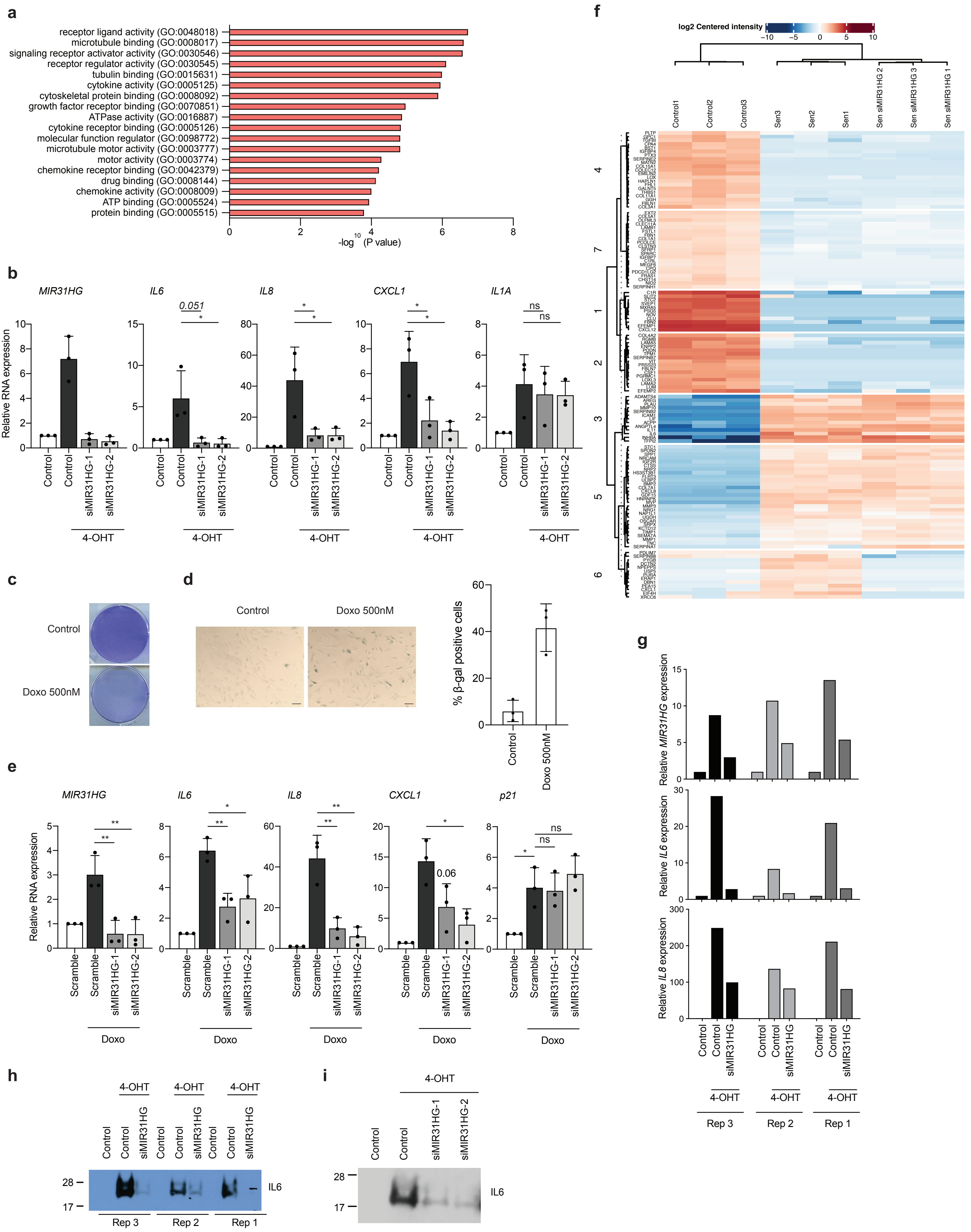

Supplementary Figure 1 (**a**) GO enrichment analysis for the significant downregulated genes in BJ ER:BRAF senescent *MIR31HG* knock-down cells compared to control knock-down senescent cells treated with 1 μM 4-OHT for 48 h. The graph displays the molecular function of the significant categories. **(b)** qRT–PCR analysis of selected components of the SASP normalized to housekeeping genes (*HPRT1* and *RPLP0*) in TIG3 ER:BRAF cells transfected with the indicated siRNAs (Control or siMIR31HG1-2), treated with ethanol (Control) or 1 μM 4-OHT for 48 h. The graphs show results compared to control ethanol-treated set to 1 (n=3). **(c)** Representative image of a crystal violet staining experiment in BJ cells treated with DMSO (control) or with 500 nM doxorubicin for 48 h (n=2). **(d)** Left, representative images of the β-galactosidase staining in BJ cells treated with DMSO (control) or with 500 nM doxorubicin for 48 h (n=3). Scale bar: 50 μm. Right, quantification of the percentage of β-galactosidase positive cells in BJ control or treated with 500 nM doxorubicin for 48 h. Error bars represent mean ± s.d. (n=3). **(e)** qRT–PCR analysis of selected components of the SASP normalized to housekeeping genes (*HPRT1* and *RPLP0*) in BJ cells transfected with the indicated siRNAs (Control or si*MIR31HG*1-2), treated with DMSO (Control) or 500 nM doxorubicin for 48 h. The graphs show results compared to control DMSO-treated set to 1 (n=3). **(f)** The heat map represents the intensity of all the proteins identified by mass spectrometry described in Fig. 1c. The heat map shows BJ ER:BRAF ethanol treated cells (Control 1-3), BJ ER:BRAF transfected with control siRNA (Sen 1-3) or siMIR31HG (Sen siMIR31HG 1-3) treated with 1 μM 4-OHT for 72 h. (**f**) BJ ER:BRAF (Control or siMIR31HG1-2) were treated for 72 h with 1 μM 4-OHT. The CM was then harvested and proteins were precipitated using EtOH. IL6 protein expression was analysed by western blot. Molecular weight marker is shown in kDa (n=3). All error bars represent means ± s.d. **(g)** qRT–PCR analysis of total RNA extracted from the cells that were subjected to mass spectrometry described in Fig. 1c. **(h)** Western blot analysis for IL6 secretion from precipitated protein using EtOH precipitation method from the CM that was subjected to mass spectrometry described in Fig. 1c. The blot shows the results from 3 independent experiments. Molecular weight marker is shown in kDa. **(i)** Validation of the decreased secretion of IL6 by western blot of precipitated proteins using two different siRNAs against MIR31HG (n=3)**.** All statistical significances were calculated using two-tailed Student t-tests. *p< 0.05; **p < 0.01; ns, non-significant.

Supplementary Figure 2

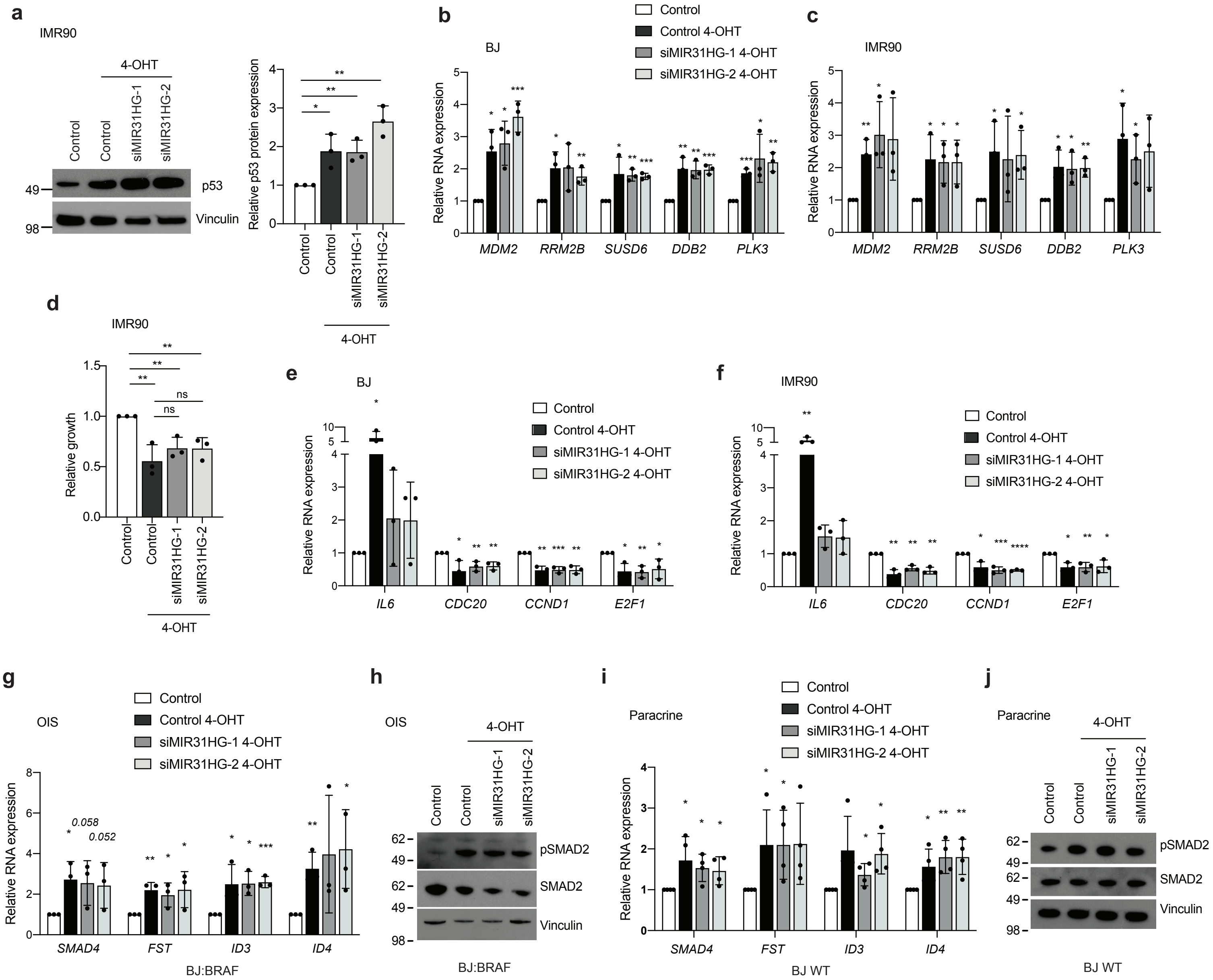

Supplementary Figure 2 **(a)** IMR90 cells were incubated for 72 h with the CM collected from BJ ER:BRAF cells (Control or si*MIR31HG*1-2) treated with ethanol (Control) or 1 μM 4-OHT for 72 h. *Left*, whole cell extracts were analysed by western blot for p53 and Vinculin. Molecular weight marker is shown in kDa. *Right*, quantification of the intensity of the bands using Fiji software (n=3). **(b-c)** qRT–PCR analysis of selected p53 target genes relative to housekeeping genes (*HPRT1* and *RPLP0*) in wild type BJ (b) and IMR90 (c) cells were incubated for 72 h with the CM collected from BJ ER:BRAF cells (Control or si*MIR31HG*1-2) treated with ethanol (Control) or 1μM 4-OHT for 72 h (n=3). **(d)** IMR90 cells were incubated for 72 h with the CM collected from BJ ER:BRAF cells (Control or si*MIR31HG*1-2) treated with ethanol (Control) or 1 μM 4-OHT for 72 h. The number of cells was measured by crystal violet staining dissolved in acetic acid and measure at 590 nm. The graph shows the absorbance relative to the control cells set as 1 (n=3). **(e-f)** qRT–PCR analysis of a subset of cell-cycle related genes normalized to housekeeping genes (*HPRT1* and *RPLP0*) in total RNA extracted from cells described in (a-b). The graph shows the RNA expression relative to control cells set to 1 (n=3). **(g)** qRT–PCR analysis of selected TGFβ target genes relative to housekeeping genes (*HPRT1* and *RPLP0*) in BJ ER:BRAF cells transfected with the indicated siRNAs (Control or siMIR31HG1-2), treated with ethanol (Control) or 1 μM 4-OHT for 48 h. The graph shows results compared to control ethanol-treated set to 1 (n=3). **(h)** Western blot for p-SMAD and total SMAD in the cells described in (b). Vinculin was used as loading control. Molecular weight marker is shown in kDa (n=2). **(i)** qRT–PCR analysis of selected TGFβ target genes relative to housekeeping genes (*HPRT1* and *RPLP0*) in BJ wild type (BJ WT) cells that have been incubated with the CM from cells described in (a) for 72h (n=3). **(j)** Western blot for p-SMAD and total SMAD in the cells described in (c). Vinculin was used as loading control. Molecular weight marker is shown in kDa (n=2). All statistical significances were calculated using two-tailed Student t-tests, *$p < 0.05$; **$p < 0.01$; ***$p<0.001$. All error bars represent means ± s.d.

Supplementary Figure 3

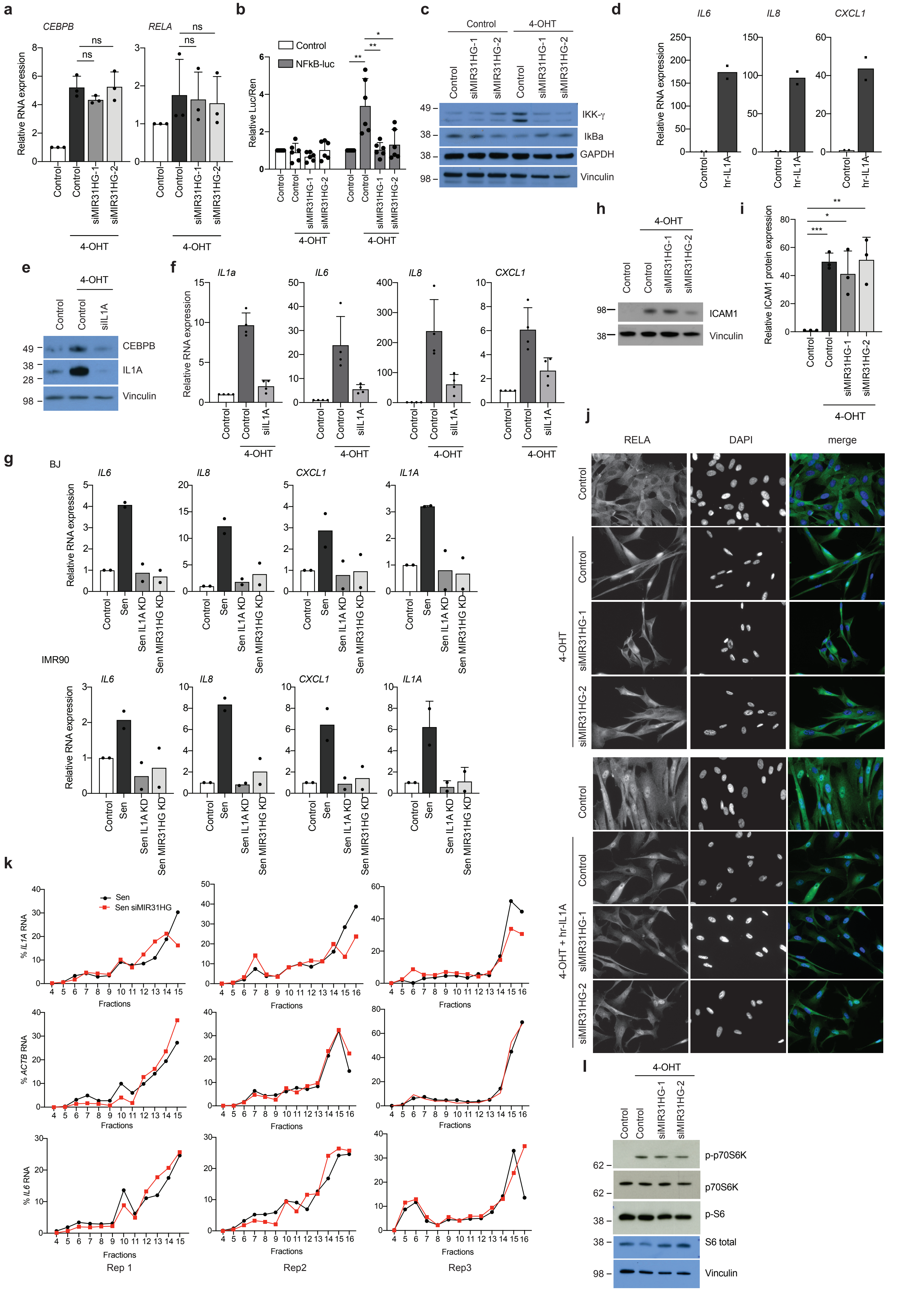

Supplementary Figure 3 **(a)** qRT-PCR analysis of *CEBPB* and *RELA* mRNA normalized to the housekeeping genes (HPRT1 and RPLP0) in BJ ER:BRAF cells (Control or siMIR31HG1-2) treated with ethanol (Control) or 1 μM 4-OHT for 48 h. The graph shows relative RNA expression to control untreated cells set to 1 (n=3). **(b)** Luciferase expression in BJ-BRAF cells transfected with luciferase reporter constructs pGL3-promoter (Control) or 4 NF-kB-luc and with the indicated siRNAs in Control or 4-OHT-treated cells. The graph shows the firefly luciferase relative to renilla units from 6 experiments. **(c)** BJ ER:BRAF cells transfected with the indicated siRNAs (Control or siMIR31HG1-2), treated with ethanol (Control) or 1 μM 4-OHT for 72h were analysed by western blot for p-RELA, CEBPB, IKK-γ and IKBa. Vinculin is shown as loading control. Molecular weight marker is shown in kDa (n=2). **(d)** qRT-PCR analysis of a subset of SASP components in BJ ER:BRAF cells untreated or treated with 10 ng/ml of human recombinant IL1A (hr-IL1A) for 2h (n=2). **(e)** BJ ER:BRAF (Control or siIL1A) treated with ethanol (Control) or 1 μM 4-OHT for 72h were analysed by western blot for CEBPB, IL1A and Vinculin. Molecular weight marker is shown in kDa (n=3). **(f)** qRT-PCR analysis of a subset of SASP components in the conditions described in (c) (n=5). **(g)** Wild type BJ (top) and IMR90 (bottom) cells were incubated for 72 h with the CM collected from BJ ER:BRAF cells (Control, siIL1A or si*MIR31HG*) treated with ethanol (Control) or 1 μM 4-OHT for 72 h. The graphs show the RNA expression of SASP components relative to housekeeping genes (*HPRT1* and *RPLP0* relative to control ethanol-treated cells set to 1 (n=2). **(h)** Western blot analysis of ICAM1 protein in control or *MIR31HG*-depleted cells. **(i)** Quantification of the density of ICAM1 bands related to GAPDH from (n=3)**.** (**j**) Immunofluorescence for RELA and DAPI staining (n=2) in BJ ER:BRAF cells transfected with the indicated siRNAs (Control or siMIR31HG1-2), treated with ethanol (Control) or 1 μM 4-OHT for 48 h, in the absence or presence of 10ng/ml for 24h before fixation. **(k)** Distribution of *ILA*, *ACTB* and *IL6* mRNA in the three polysome fractionations performed by sucrose gradient in BJ ER:BRAF control cells (Sen, black) or siMIR31HG cells (Sen MIR31HG KD, red) treated with 1 μM 4-OHT for 72 h. **(l)** BJ ER:BRAF cells transfected with the indicated siRNAs (Control or siMIR31HG1-2), treated with ethanol (Control) or 1μM 4-OHT for 72h were analysed by western blot for p-p70S6K, p70S6K, p-S6, S6 and Vinculin. Molecular weight marker is shown in kDa (n=3). Statistical significances were calculated using two-tailed Student t-tests, *p < 0.05; **p< 0.01; ***p < 0.005. All error bars represent means ± s.d.

Supplementary Figure 4

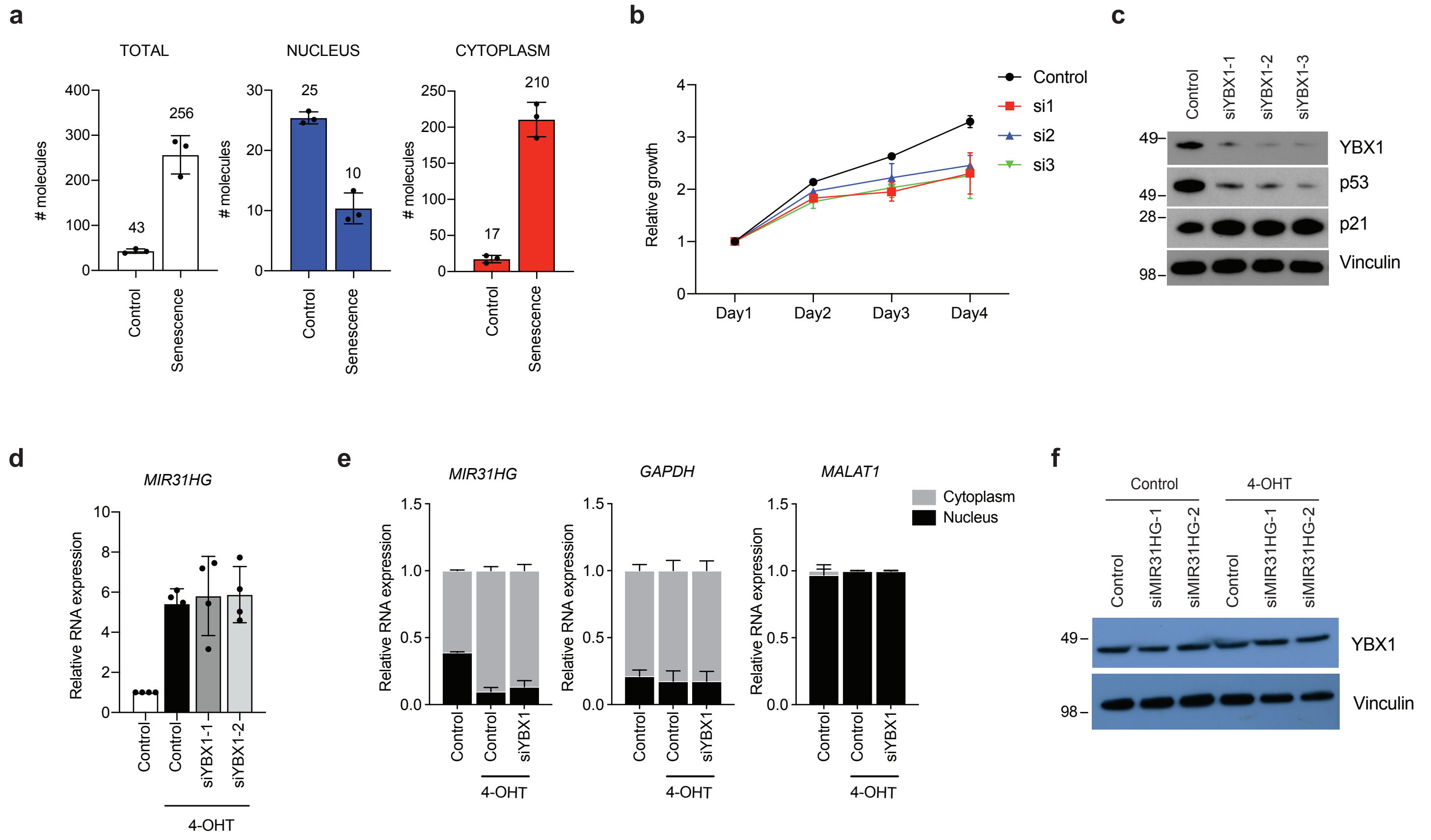

Supplementary Figure 4 (a) Number of molecules of *MIR31HG* after cellular fractionation in BJ ER:BRAF control or 4-OHT-treated cells, calculated by q-PCR using an in vitro transcribed *MIR31HG* as template. (**b)** BJ ER:BRAF (control or siYBX1-1-3) were stained with crystal violet staining (see materials and methods) to address the cell growth at the indicated days post-transfection. The graph shows the absorbance (590 nM) measured after dissolving the crystal violet in 10% acetic acid (n=3). **(c)** BJ ER:BRAF (control or siYBX1-1-3) were analysed for western blot 72 h post-transfection for YBX1, p53, p16 and Vinculin. Molecular weight marker is shown in kDa (n=2). **(d)** BJ ER:BRAF cells transfected with the indicated siRNAs (control or siYBX1-1-2), treated with ethanol (Control) or 1 μM 4-OHT for 48 h were analysed by qRT–PCR for *MIR31HG* mRNA relative expression normalized to housekeeping genes (HPRT1 and RPLP0) and represented relative to untreated control cells (n=4). **(e)** BJ ER:BRAF (control or siYBX1) treated with ethanol (Control) or 1μM 4-OHT for 48 h were fractionated and the RNA was extracted. Distribution of *MIR31HG*, *GAPDH* and *MALAT1* (nuclear (grey), cytoplasmic (black)) was analysed by qRT-PCR. The graph shows the percentage of transcript relative to the input (n=2). **(f)** BJ ER:BRAF cells transfected with the indicated siRNAs (Control or siMIR31HG1-2), treated with ethanol (Control) or 1 μM 4-OHT for 72 h were analysed by western blot for YBX1 total levels. Molecular weight marker is shown in kDa. All error bars represent means ± s.d.

Supplementary Figure 5

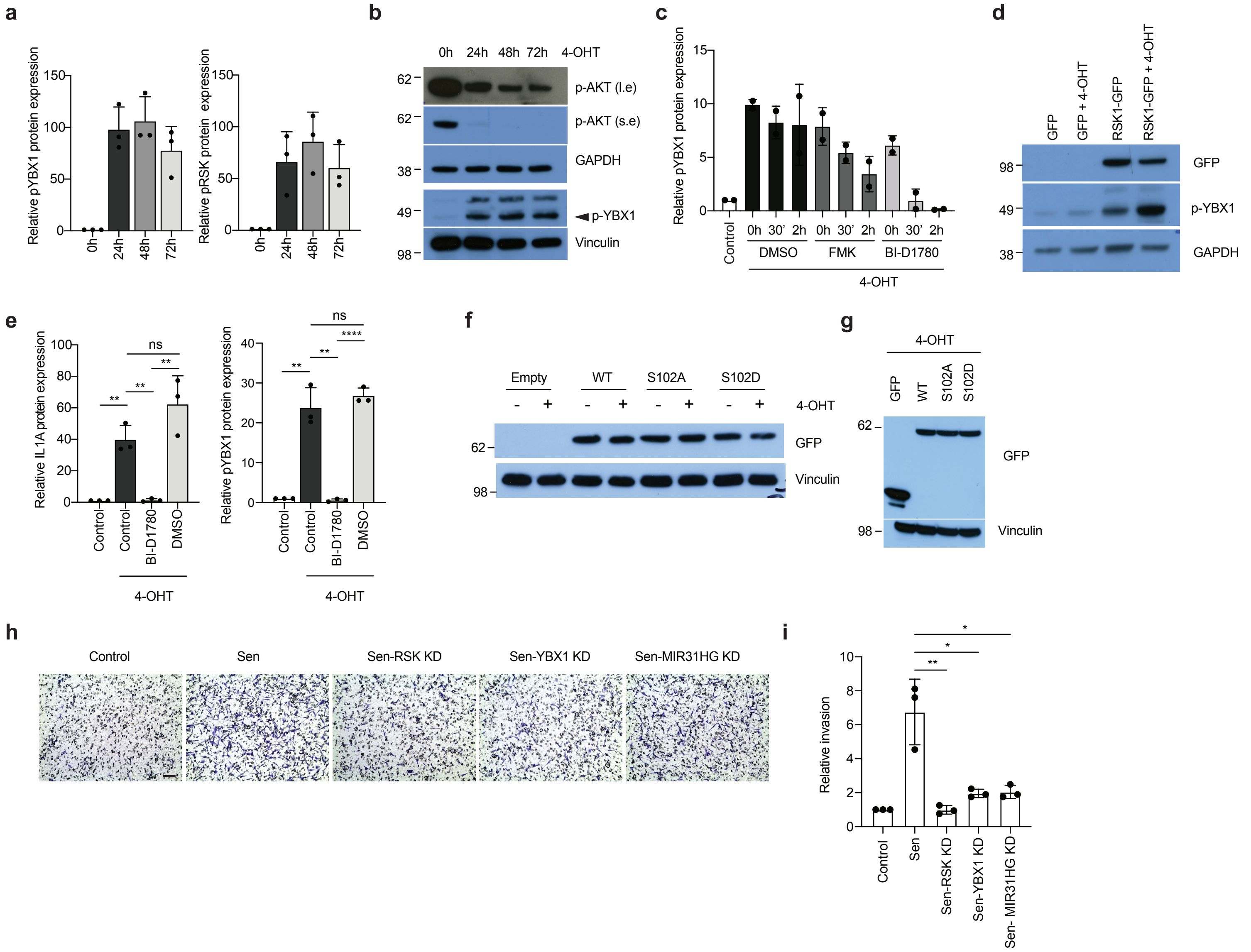

Supplementary Figure 5 (a) Quantification from the western blot in Fig. 5a, of the pYBX1 and p-RSK band intensities relative to total YBX1 and total RKS respectively and to Vinculin (n=3). **(b)** Western blot for p-AKT long exposure (l.e) or short exposure (s.e) and pYBX1 (arrow) in BJ ER:BRAF cells treated with 4-OHT for the indicated time. GAPDH and Vinculin were used as loading control. Molecular weight marker is shown in kDa (n=2). **(c)** Quantification of the pYBX1 band intensities relative to total YBX1 and Vinculin from Fig. 5b (n=3). **(d)** Western blot analysis for GFP, p-YBX and YBX1 in empty-GFP BJ ER:BRAF or RSK1-GFP BJ ER:BRAF induced with doxycycline (empty: 20 ng/ml, RSK1: 100 ng/ml) untreated or treated with 1 μM 4-OHT for 72 h (n=3). **(e)** Quantification of the p-YBX1, total YBX1 and IL1A band intensities relative to Vinculin from Fig. 5c (n=3). **(f)** Western blot analysis to control the expression of the GFP-tagged proteins from experiment in Fig 5d (n=3). **(g)** Western blot analysis for GFP to control the expression of the GFP-tagged proteins from experiment in Fig. 5e (n=3). **(h)** MDA-MB-231 invading cells through a matrigel membrane in contact with the CM from BJ ER:BRAF cells transfected with the indicated siRNA and treated for 72 h with ethanol (Control) or 1 μM 4-OHT. Representative images are shown in the figure (n=3). Scale bar: 50 μm **(i)** Quantification of the invading cells from (h) relative to control ethanol-treated cells (n=3). All statistical significances were calculated using two-tailed Student t-tests, *$p < 0.05$; **$p < 0.01$. All error bars represent means ± s.d.

Supplementary Figure 6

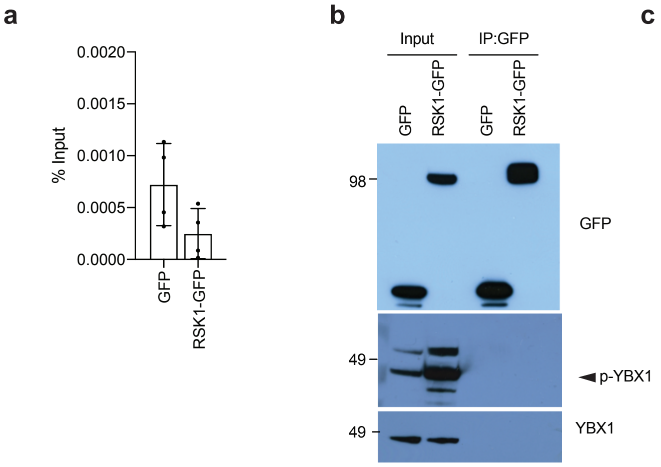

Supplementary Figure 6 (a) Native RNA-IP experiment of empty-GFP BJ ER:BRAF or RSK1-GFP BJ ER:BRAF induced with doxycycline (empty:20ng/ml, RSK1: 100ng/ml) and treated with 1 μM 4-OHT for 72 h. The graph shows the percentage of the input of *MIR31HG* bound to RSK (n=3). **(b)** Western blot analysis for GFP, p-YBX and YBX1 to control the IP efficiency in (a). **(c)** *In vitro* kinase experiment incubating recombinant YBX1 in the presence of ^{32}P-ATP and the indicated ng of recombinant RSK. The reaction was run in 4–12% NuPAGE Bis–Tris gel and exposed to an Amersham Hyperfilm ECL film. The image shown a representative experiment (n=3). **(d)** qRT–PCR analysis for *MIR31HG* expression levels of the doxycycline-inducible cell lines overexpressing empty construct, *MIR31HG* WT, Mut3 and Mut5 untreated or treated with doxicyclin for 48h. The graph shows *MIR31HG* expression compared to an empty cell line set to 1 (n=2). All error bars represent means ± s.d.

Supplementary Table 1. List of primers and oligonucleotides sequences

qPCR

Gene	**FORWARD**	**REVERSE**
MIR31HG	CGCTTCTGTCCTCCTACTCG	ACAAGCAGACCCTTGGAATG
HPRT1	GTAATTGGTGGAGATGATCTCTCAACT	TGTTTTGCCAGTGTCAATTATATCTTC
GAPDH	ATG GGG AAG GTG AAG GTC G	GGG TCA TTG ATG GCA ACA ATA TC
RPLP0	TTCATTGTGGGAGCAGAC	CAGCAGTTTCTCAGAGC
CXCL1	GCGCAATCCAGGTGGCCTCT	GCCTCCTTCAGGAACAGCCACCA
CXCL2	GGGCAGAAAGCTTGTCTCAACCCC	GCGCAATCCAGGTGGCCTCT
IL6	GCACTGGCAGAAAACAACCT	CAGGGGTGGTTATTGCATCT
IL8	CCAGGAAGAAACCACCGGAA	CTCCTTGGCAAAACTGCACC
IL1A	ACCTCACGGCTGCTGCATTACA	TCCTTCAGCAGCACTGGTTGGT
ICAM1	GAACCAGAGCCAGGAGACAC	CTTCACTGTCACCTCGGTCC
CEBPB	AAGCACAGCGACGAGTACAA	GTGAGCTCCAGGACCTTGTG
RELA	TTGAGGTGTATTTCACGGGACC	GCACATCAGCTTGCGAAAAGG
ACTB	GGCATGGGTCAGAAGGATT	GGGGTGTTGAAGGTCTCAAA
MALAT1	GAATTGCGTCATTTAAAGCCTAGTT	GTTTCATCCTACCACTCCCAATTAAT
YBX1	TCGCCAAAGACAGCCTAGAGA	TCTGCGTCGGTAATTGAAGTTG
ND1	CCCTAAAACCCGCCACATCT	GGCTAGAATAAATAGGAGGCCTAGGT
ND4	TCACAACACCCTAGGCTCACTAA	GGGAGTCATAAGTGGAGTCCGT
SMAD4	ACCCCAGCTCTGTTAGCCCCA	TGGCAGGCTGACTTGTGGAAGC
FST	GGGAGAGGCCGGTGTTCCCT	CGGTGTCTTCCGAAATGGAGTTGC
ID3	GTACCCGGAGTCCCGAGAGGC	TGAGCTCGGCTGTCTGGATGGG
ID4	ACTGCGCTCAACACCGACCC	GGCCGCACACCTGGACAGC
RRM2B	GGATCTCCCTCACTGGAACA	CGCTCCACCAAATTTTCATT
MDM2	GTATCAGGCAGGGGAGAGTG	TGTTGCAATGTGATGGAAGG
SUSD6	GCAGGATAGCACCAAAGAGC	GAAGCAAGTCCGTCTCCAAG
DDB2	GGGAACAACTAGGCTGCAAG	GTGACCACCATTCGGCTACT
PLK3	GCGCGAGAAGATCCTAAATG	TTGTCAGCGTCCTCAAAGTG
E2F1	TCGTAGCATTGCAGACCCTG	ACATCGATCGGGCCTTGTTT
CDC20	ATTCACCCAGCATCAAGGGG	AGCACACATTCCAGATGCGA
CCND1	GGCGGAGGAGAACAAACAGA	CTCCTCAGGTTCAGGCCTTG

ChIP qPCR

Gene	**FORWARD**	**REVERSE**
IL6 promoter	CGTGCATGACTTCAGCTTTAC	TGCAGCTTAGGTCGTCATTG
Chr7_unrelated	GGCAAACACCCAAGAACACT	GAGCTGGCTGTGAGAAGAGC

Cloning

	**FORWARD**	**REVERSE**
pLVX-YBX1-GFP	CCCTCGTAAAGAATTCATGAGCAGCGAGGCC GAG	ATGGATCGCGGCCGCAATCTCTCAGC CCCGCCCTGC
pLVX-RSK-GFP	CCTACCCTCGTAAAGAATTCATGCCGCTCGC CCAGCTC	ATGGATCGCGGCCGCAATCTCAGGGT GGTGGATGGCAAC
pLVX-MIR31HG	CCCTCGTAAAGAATTCAGGTTCCACGTCCGG CGCCTGGA G	GAGGTGGTCTGGATCCTTTATTGTTTT GGCAACAAAGAAGCAAGAACC
pLVX- Mut3	CCCTCGTAAAGAATTCAGGTTCCACGTCCGG CGCCTGGA G	GAGGTGGTCTGGATCCTTTATTGTTTT GGCAACAAAGAAGCAAGAACC
pLVX- Mut5	CCCTCGTAAAGAATTCAGGTTCCACGTCCGG CGCCTGGA G	GAGGTGGTCTGGATCCAGCACCAGAG AAGTTCTTTCACTAATAAC
pGEM-MIR31HG	AGGTTCCACGTCCGGC	TTT ATT GTT TTG GCA ACA AAG AAG CAA GAA CC

pGEM-MIR31HG-Mut1	TCTGCTGCATGGAACATGAAGCAGACCCTTGGAATGAA	CATGTTCCATGCAGCAGA
pGEM-MIR31HG-Mut2	TGCAGGCGGCCGCGAATTCACTAGTGATTGCATGGAACATGACCTTCC	ATCACTAGTGAATTCGCGG
pGEM-MIR31HG-Mut3	GCATGGAACATGACCTTCC	AAGGTCATGTTCCATGCATTCTCATTAGGAGACCACAAC
pGEM-MIR31HG-Mut4	TGCAGGCGGCCGCGAATTCACTAGTGATGTTGTGGTCTCCTAATGAGAA	TTCTCATTAGGAGACCACAAC
pGEM-MIR31HG-Mut5	TGCAGGCGGCCGCGAATTCACTAGTGATAGCACCAGAGAAGTTCTTTC	ATCACTAGTGAATTCGCGG
	mutagenesis	
	**FORWARD**	**REVERSE**
YBX1-S102A	GGAAGTACCTTCGCgcaGTAGGAGATGGAG	CTCCATCTCCTACtgcGCGAAGGTACTTCC
YBX1-S102D	GGAAGTACCTTCGCgacGTAGGAGATGGAG	CTCCATCTCCTACgtcGCGAAGGTACTTCC
	T7 promoter	
	**FORWARD**	**REVERSE**
T7-ND4	GGATCCTAATACGACTCACTATAGATGCTAAAACTAATCGTCCCAACAATT	AAGAGGAAAACCCGGTAATGATG

Luciferase reporter

	**FORWARD**	**REVERSE**
pGL3-IL1A_3UTR	CCTCTAGAGTCTGGAGTCTCACTTGTC	CCTCTAGATGTCAGAGAATTTTGTTGCAAGC

siRNAs

**RNA**	**SENSE**	**ANTISENSE**
MIR31HG-1	AAGAAUGUGUUGUGGACACAA	UUGUGUCCACAACACAUUCUU
MIR31HG-2	AAUGGAGCACAAAUAGUUU	AAACUAUUUGUGCUCCAUU
YBX1-1	UGACACCAAGGAAGAUGUA	UACAUCUUCCUUGGUGUCA
YBX1-2	GGUUCCCACCUUACUACAU	AUGUAGUAAGGUGGGAACC
YBX1-3'UTR	CUUACCAUCUCUACCAUCA	UGAUGGUAGAGAUGGUAAG
IL1A	GCCCUCAAUCAAAGUAUAAUU	AAUUUAUACUUUGAUUGAGGGC
	**Product**	**Reference**
RSK1	Silencer™ Pre-Designed RPS6KA1 siRNA	AM51331 siRNA ID 354
RSK2	Silencer™ Pre-Designed RPS6KA3 siRNA	AM51331 siRNA ID: 553

ASOs for pulldown

**oligo_name**	**Sequence**	**Reference**
MIR31HG-1	/5AmMC12/TGTGGATGCTGATATAGAAGACA	71279986
MIR31HG-2	/5AmMC12/AATAACAGGAGGCTGGGAGGGT	71279987
MIR31HG-3	/5AmMC12/ATGGAGGGTAAAACTGGAGGC	71279988
MIR31HG-4	/5AmMC12/TAGGGAGTGACTTGATGTG	71279989
MIR31HG-5	/5AmMC12/CCAGGCTATGTCTTTCCTCTAT	71279990
**oligo_name**	**Sequence**	**Reference**
NegCtrl-1	/5AmMC12/tcactgcatacgacgattct	69313872
NegCtrl-2	/5AmMC12/attgggagcttttttgcac	69313873
NegCtrl-3	/5AmMC12/aaccgggaggtagatgagat	69313874
NegCtrl-4	/5AmMC12/agttcgcctctttgattaac	69313875
NegCtrl-5	/5AmMC12/gacgtaatccacgatctctt	69313876

Supplementary Table 2. List of Antibodies

	Company	Catalogue #	Dilution
	Western blot		
IL6	R&D Systems	AF-206-NA	1:1000
p53	santa cruz	sc-126	1:2000
VINCULIN	sigma	V 9131	1:200000
p-RELA	cell signaling	3033	1:1000
RELA	santa cruz	sc-8008	1:1000
CEBPB	santa cruz	sc-150	1:1000
GAPDH	santa cruz	25778	1:2000
IL1A for WB	abcam	ab9614	1:1000
YBX1	cell signaling	4202S	1:1000
p-YBX1 (S102)	cell signaling	2900	1:2000
p-RSK (Ser380)	cell signaling	11989S	1:5000
RSK	BD transduction labs	610225	1:2000
p-AKT	cell signaling	4060	1:1000
AKT	cell signaling	9272	1:1000
LAMIN A1	santa cruz	sc-20680	1:1000
p-SMAD2	cell signaling	3108T	1:1000
SMAD2	cell signaling	3103S	1:1000
IKK	cell signaling	2685	1:1000
IKBa	cell signaling	9242	1:1000
p-p70S6K	cell signaling	9206	1:1000
p70S6K	cell signaling	9202	1:1000
p-S6	cell signaling	2211	1:5000
S6	cell signaling	2217	1:1000
GFP	santa cruz	sc-9996	1:1000
ICAM1	cell signaling	4915S	1:2000
p21	BD Pharmigen	556431	1:2000
	IF		
YBX1	cell signaling	4202S	1:100
IL1A	R&D System	MAB200-100	1:100
	ChIP		
CEBPB	abcam	ab32358	
IgG	sigma	I8140	
	PLA		
RSK	BD transduction labs	610225	1:200
YBX1	cell signaling	4202S	1:100

Figure S1h

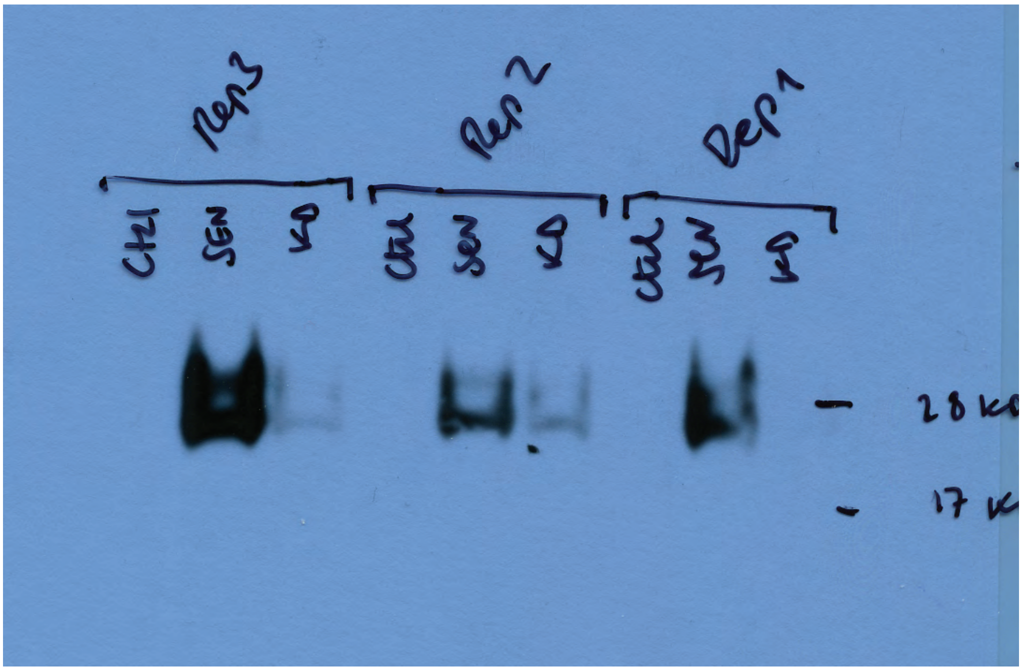

Figure S1i

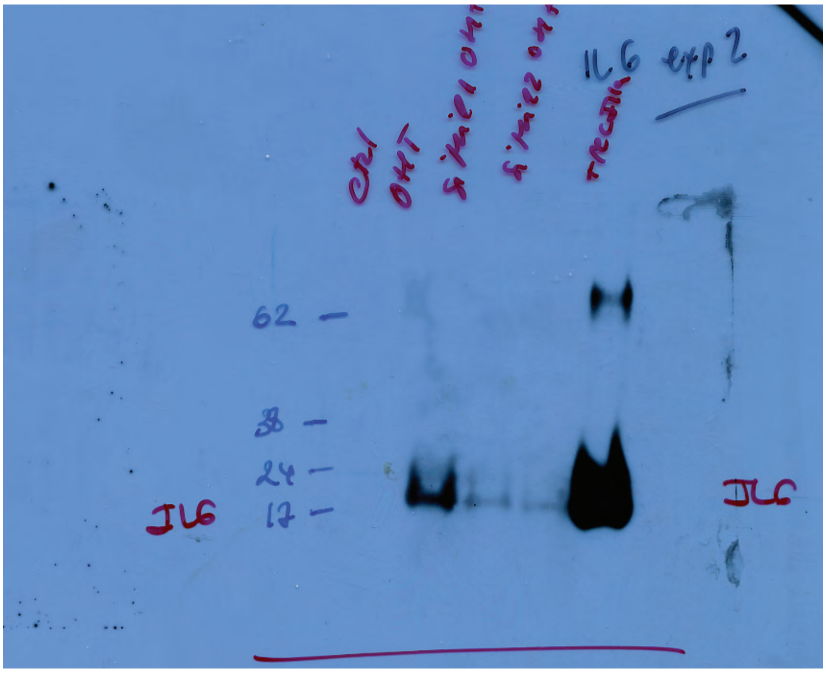

Figure 2a

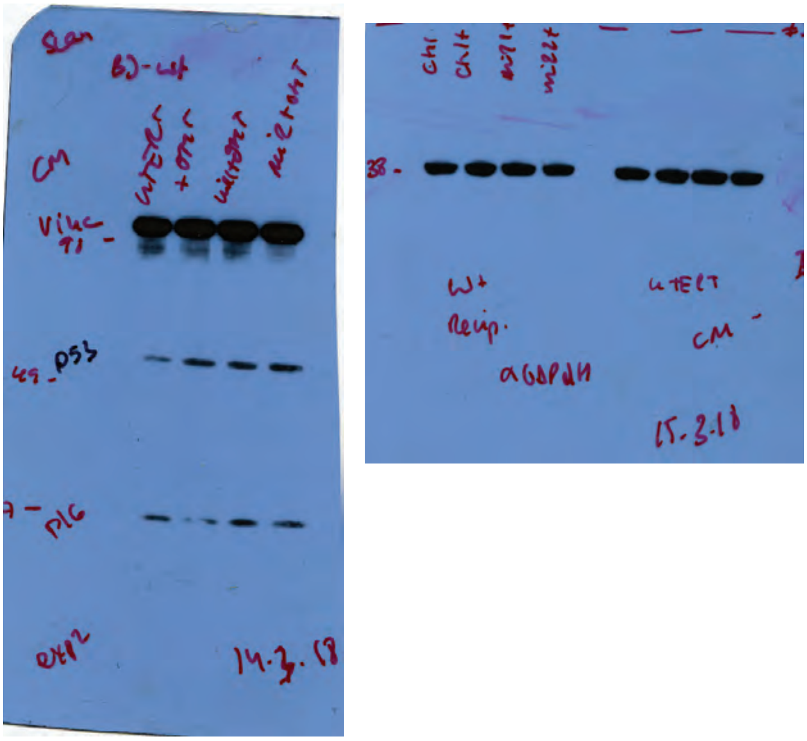

Figure S2h

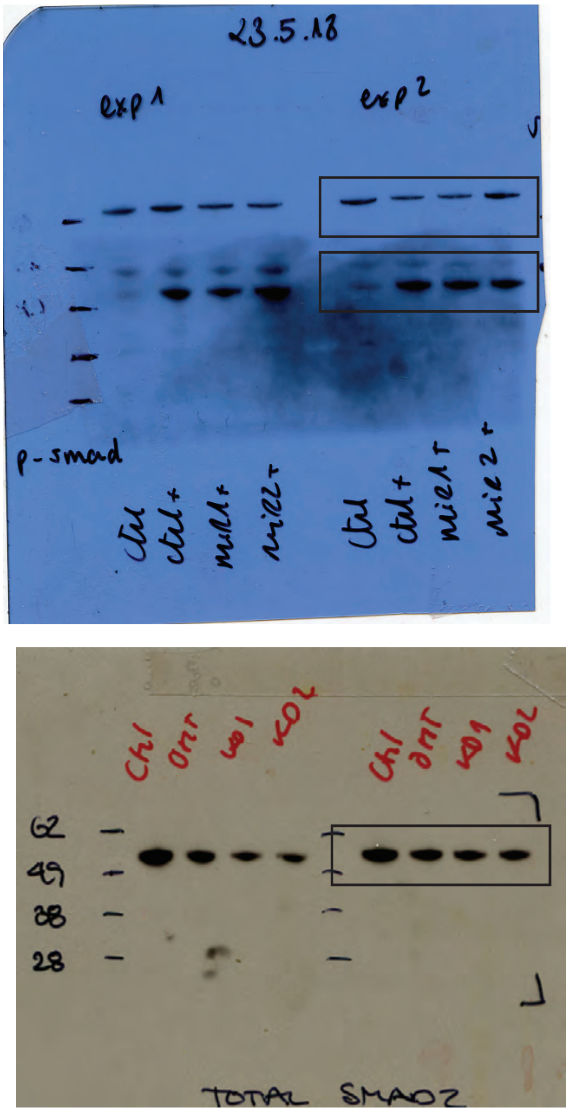

Figure S2a

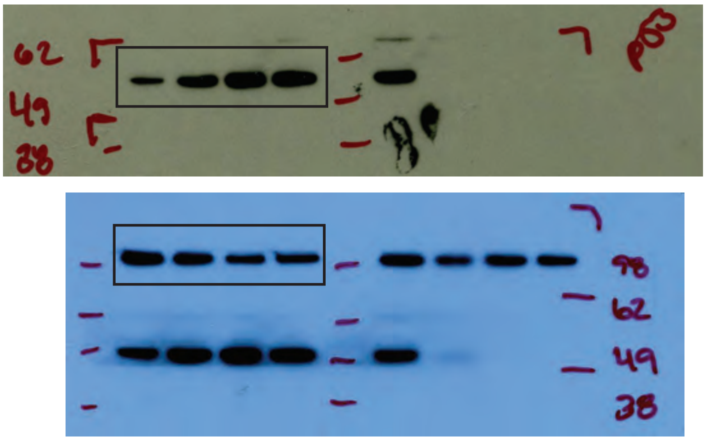

Figure S2j

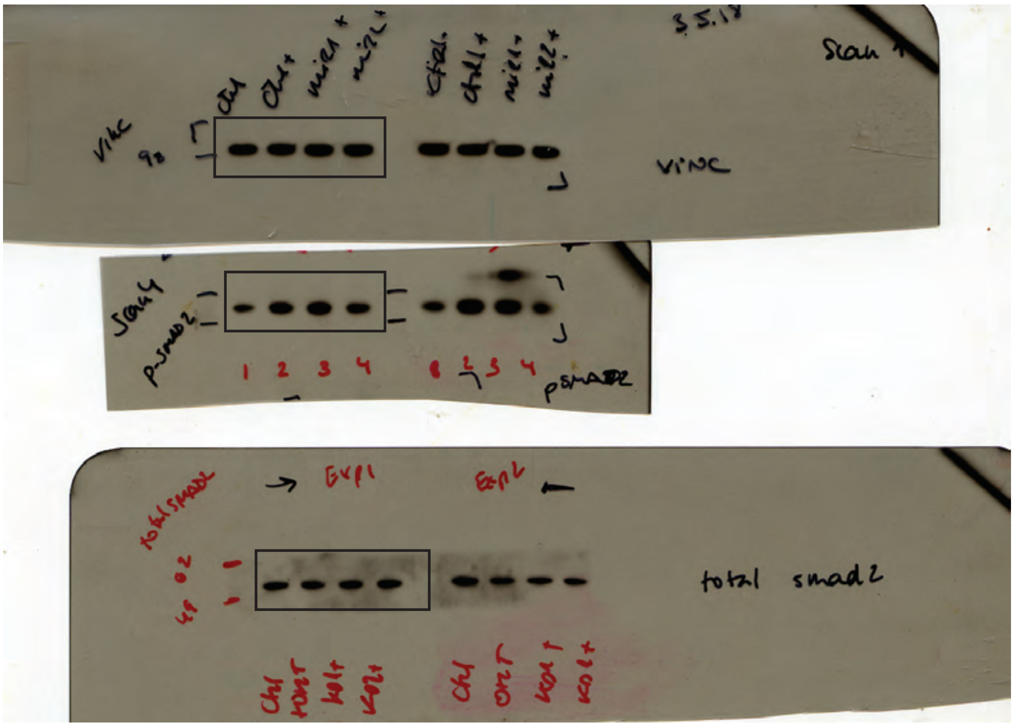

Figure 3a

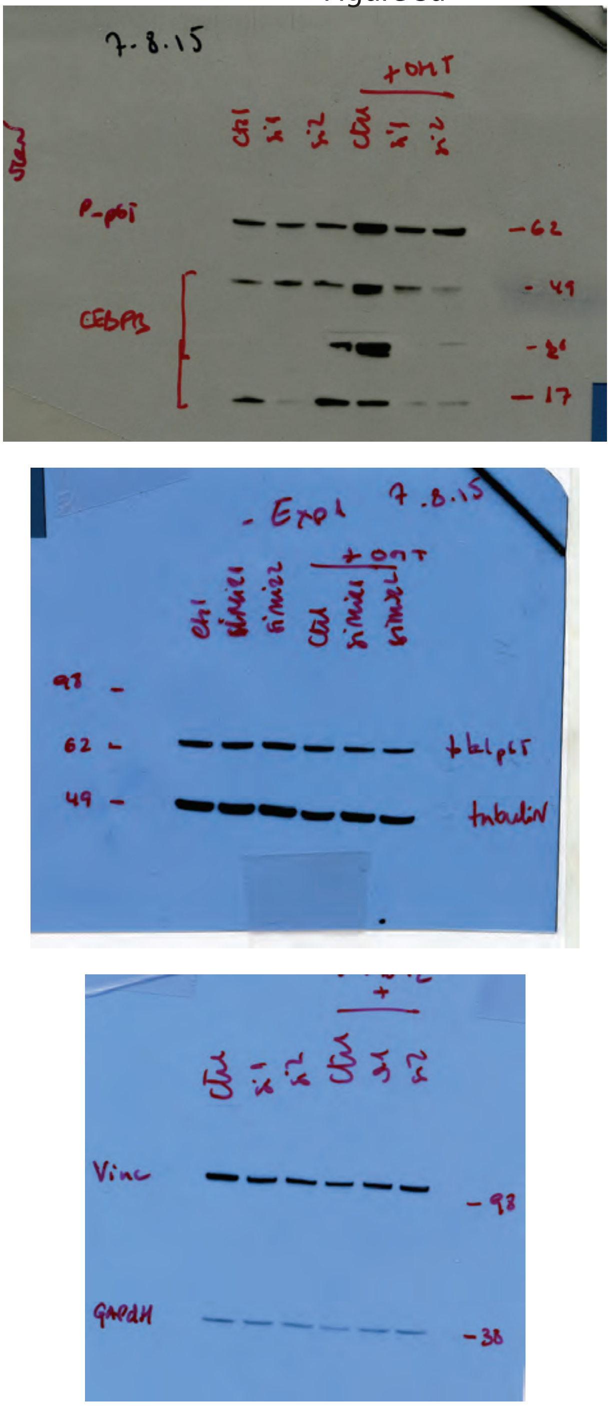

Figure 3e

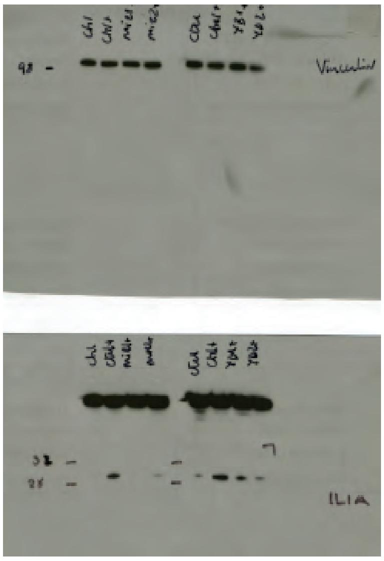

Figure 3i

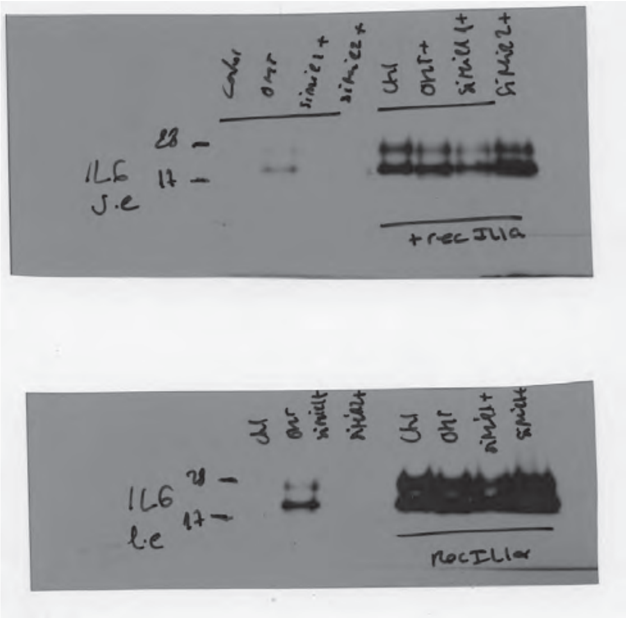

Figure 3m

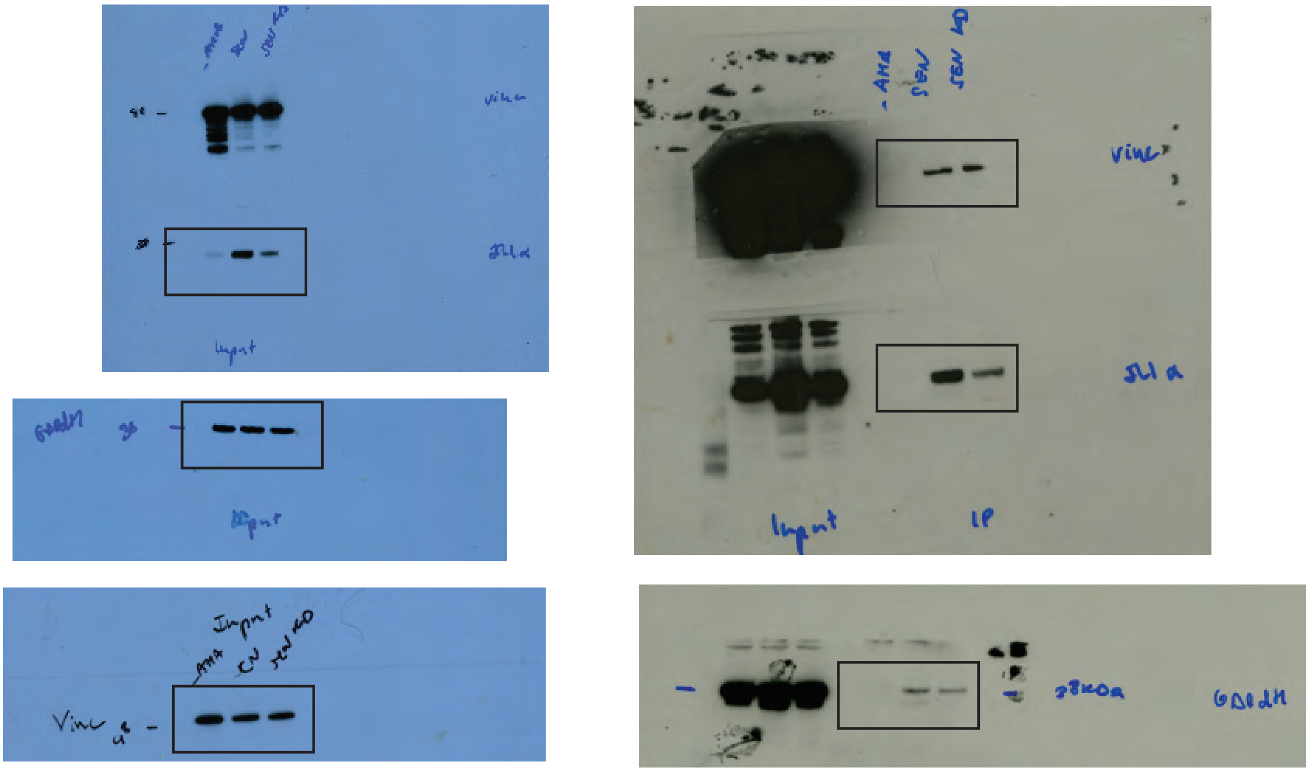

Figure S3c

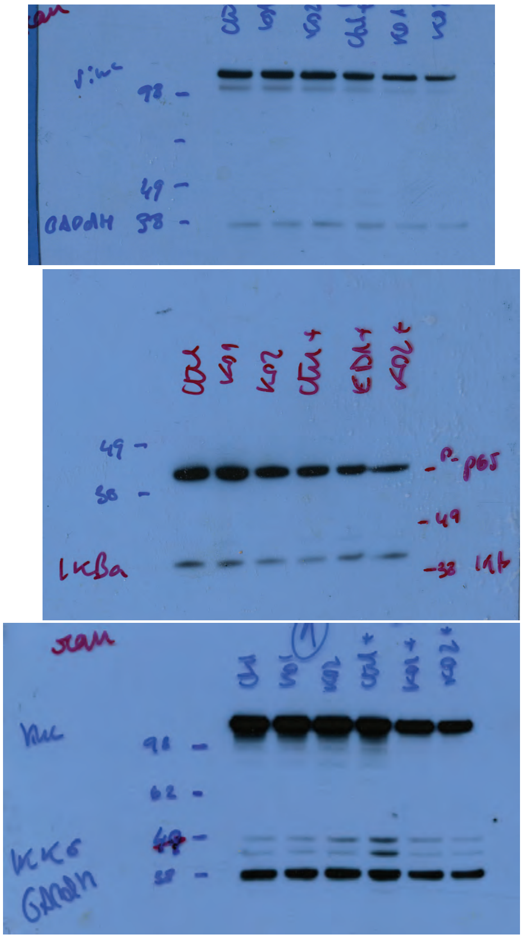

Figure S3e

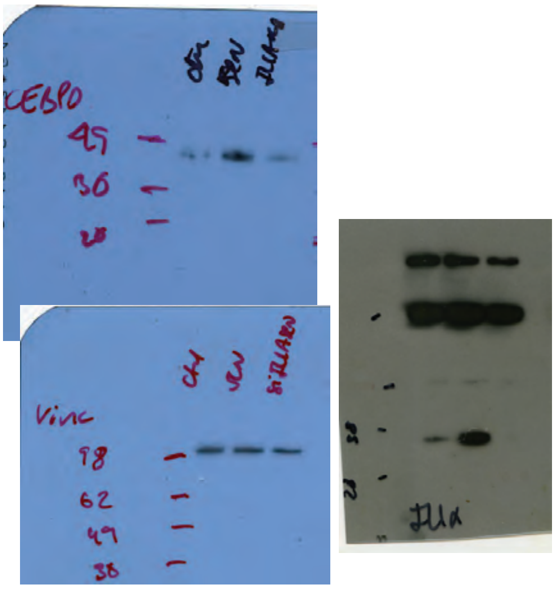

Figure S3h

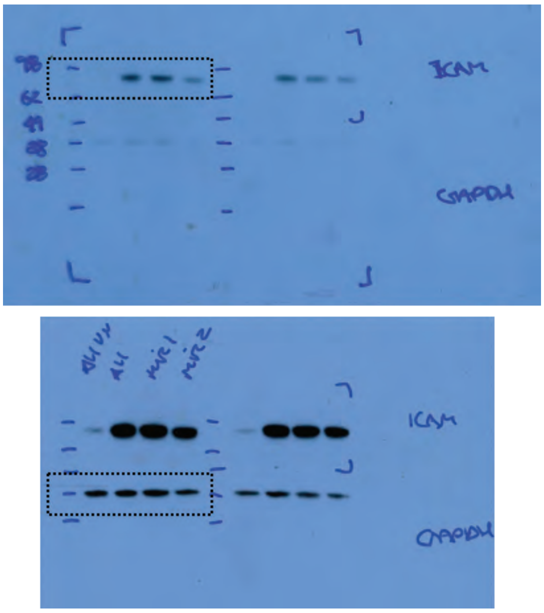

Figure S3L

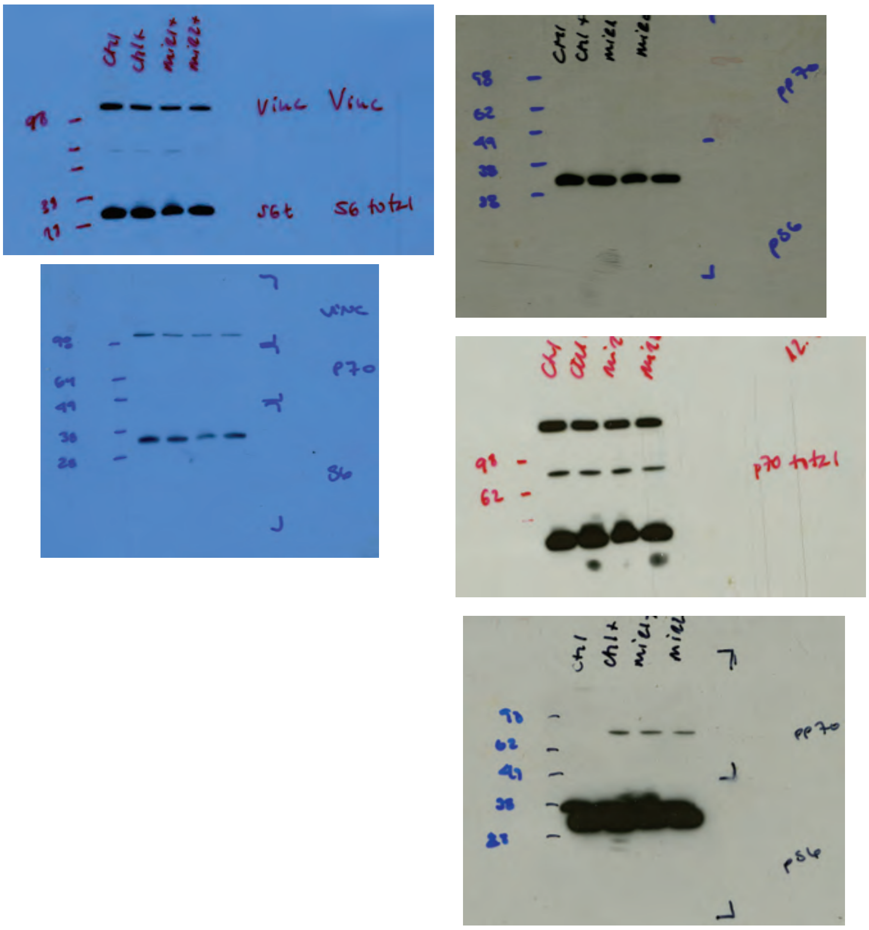

Figure 4f

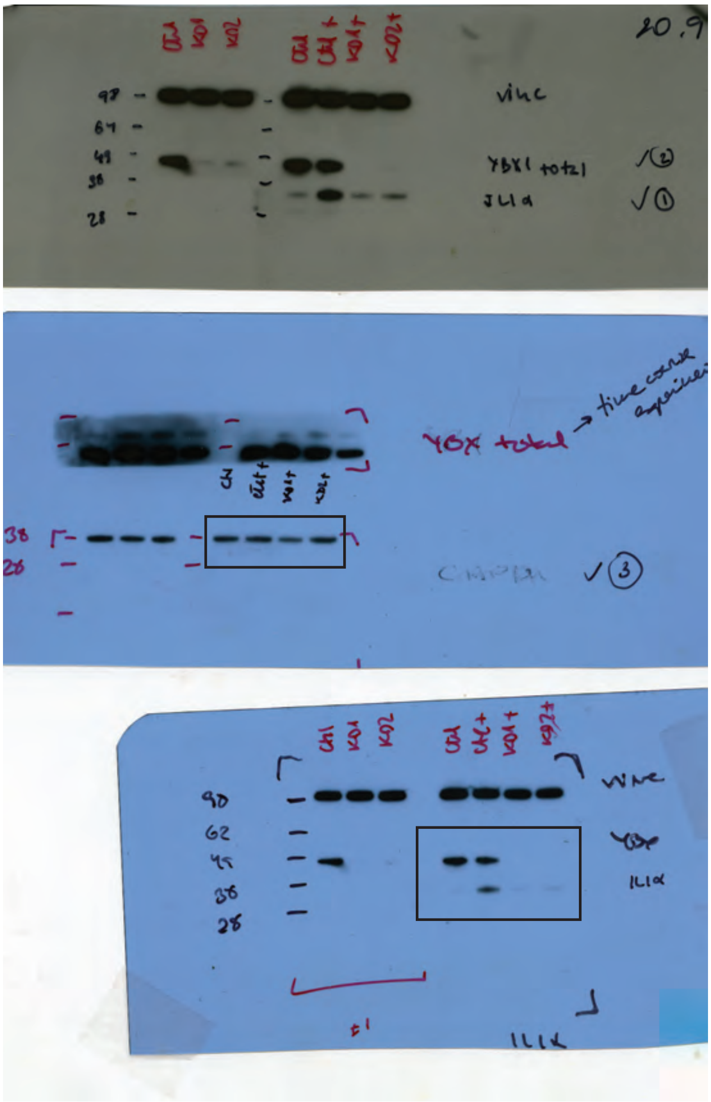

Figure 4k

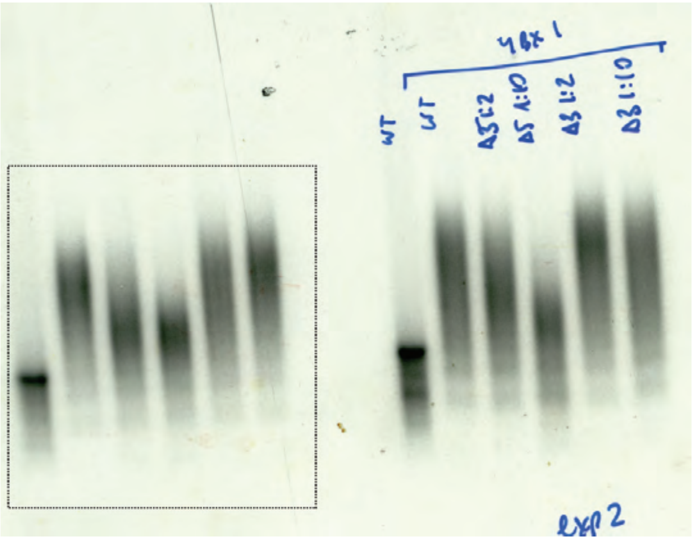

Figure 4j

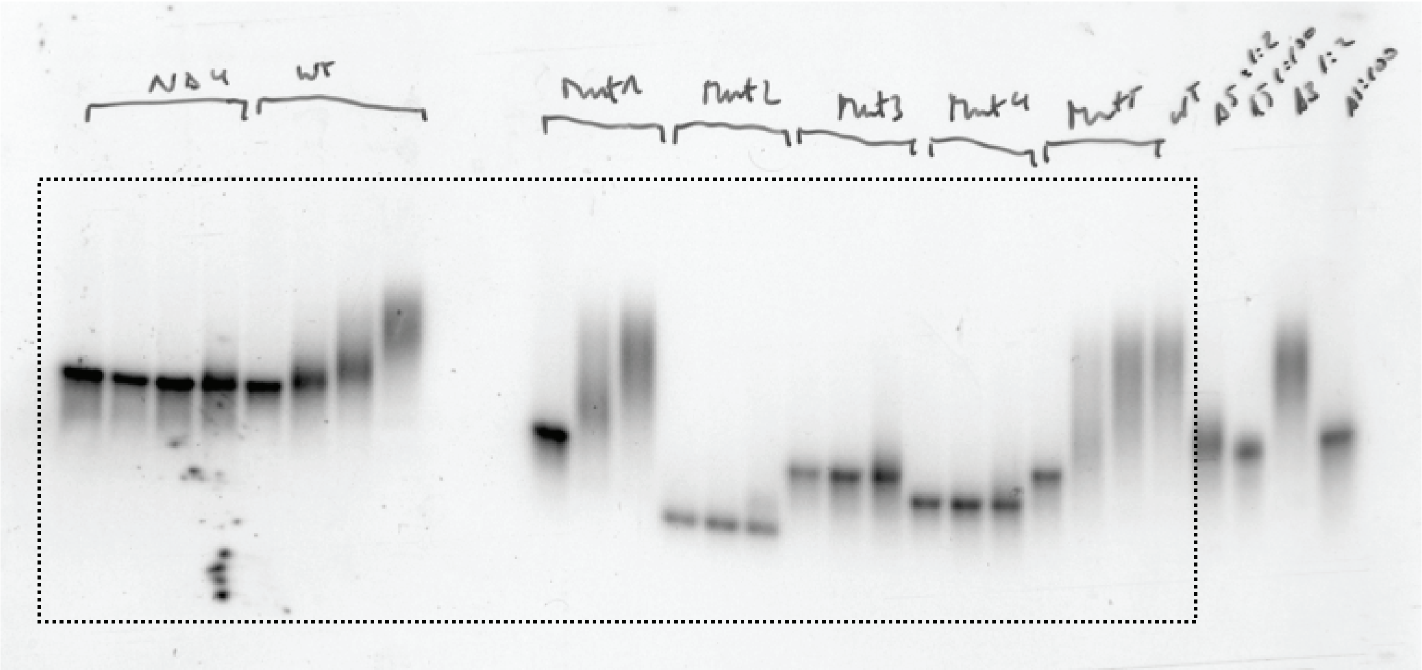

Figure S4c

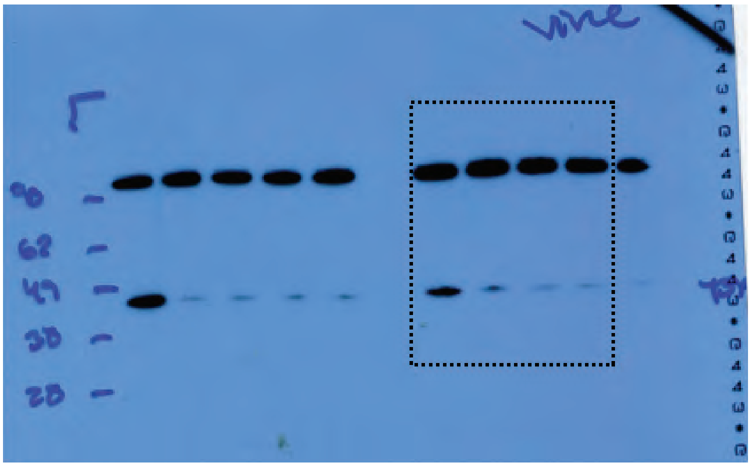

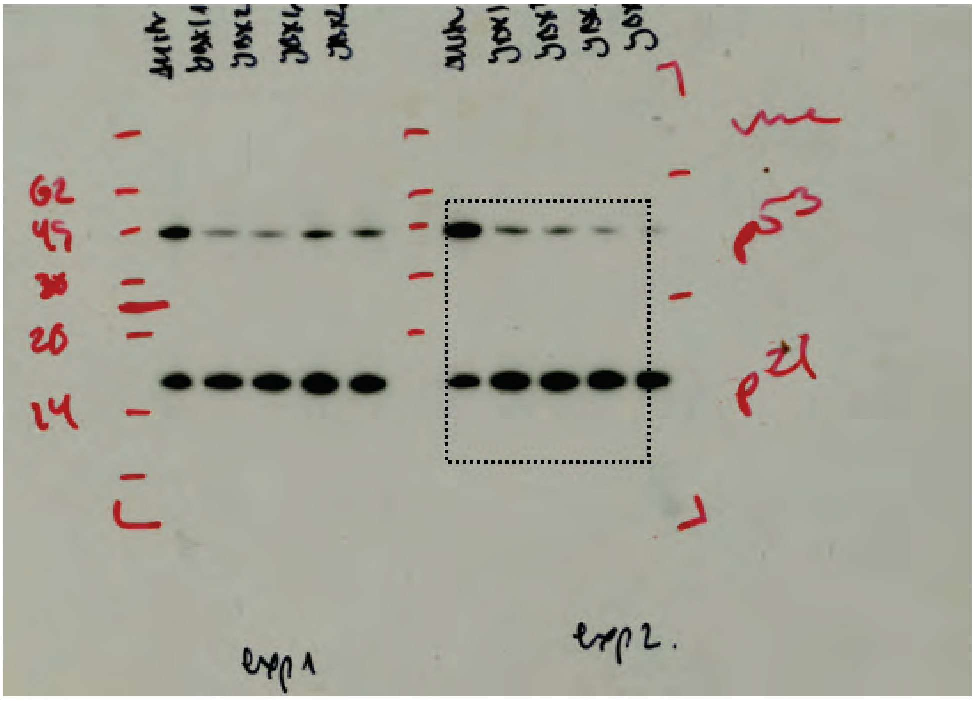

Figure S4f

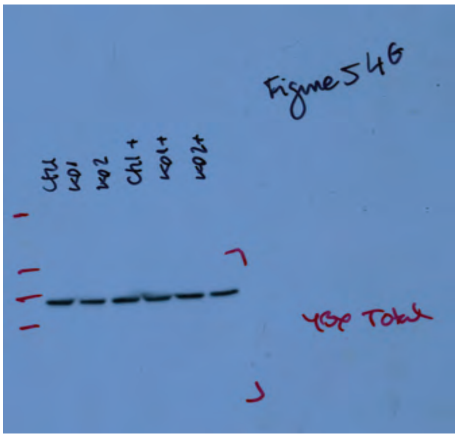

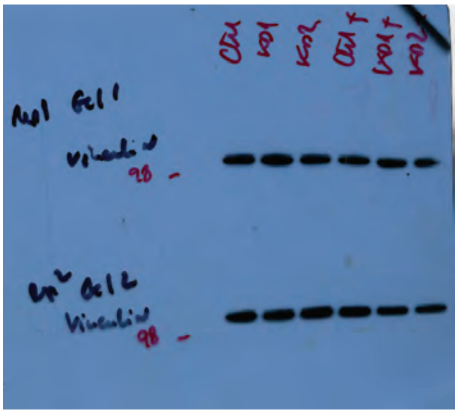

Figure 5b

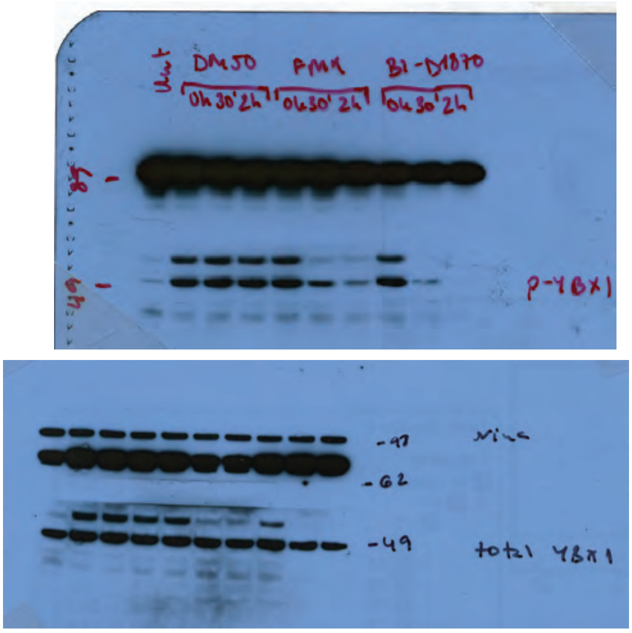

Figure 5a

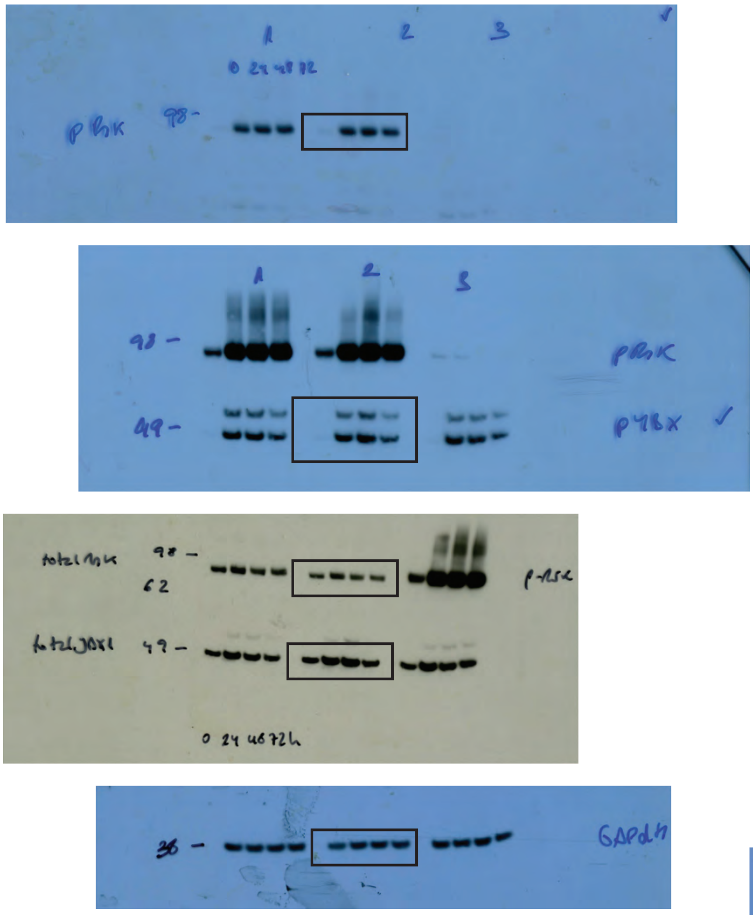

Figure 5c

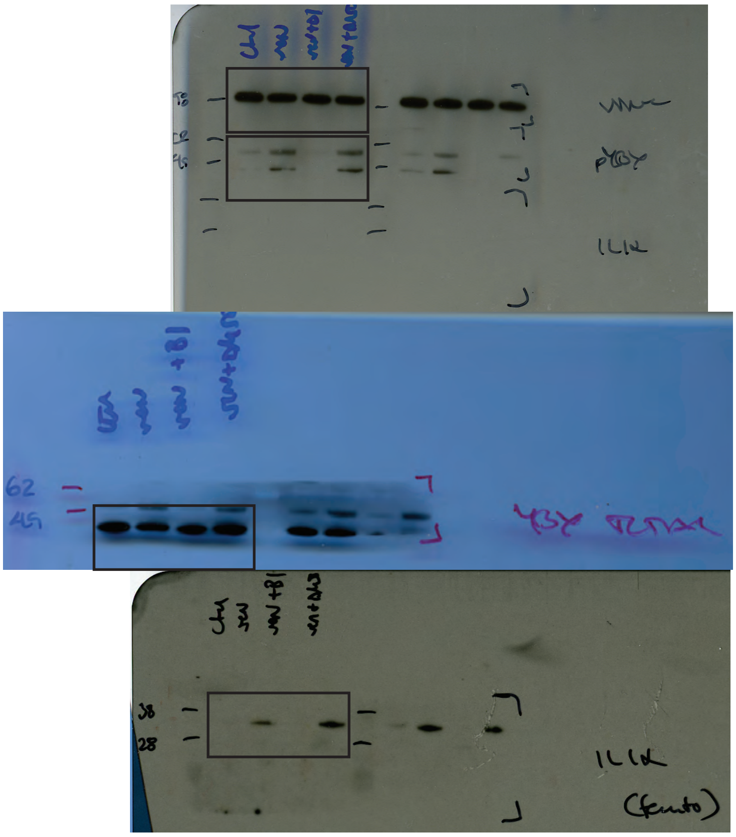

Figure S5b

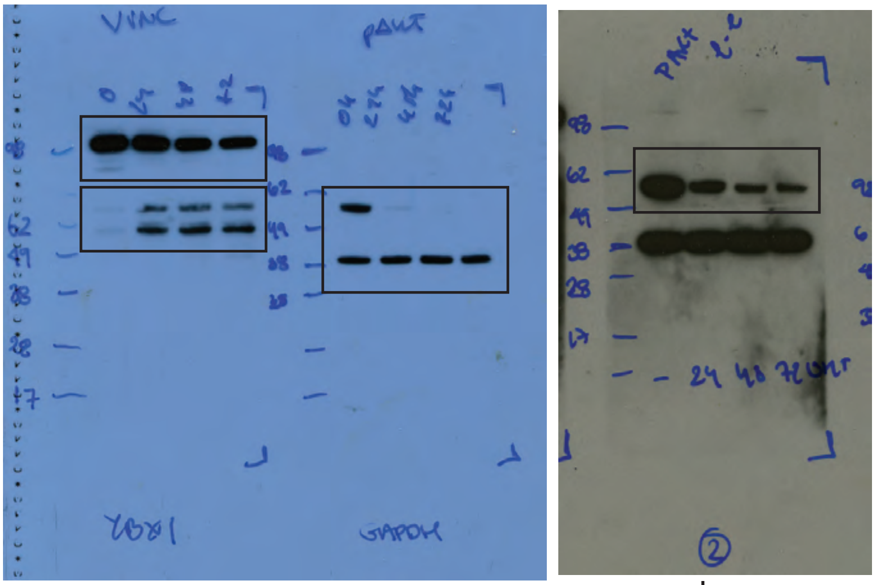

s.e

l.e

Figure S5d

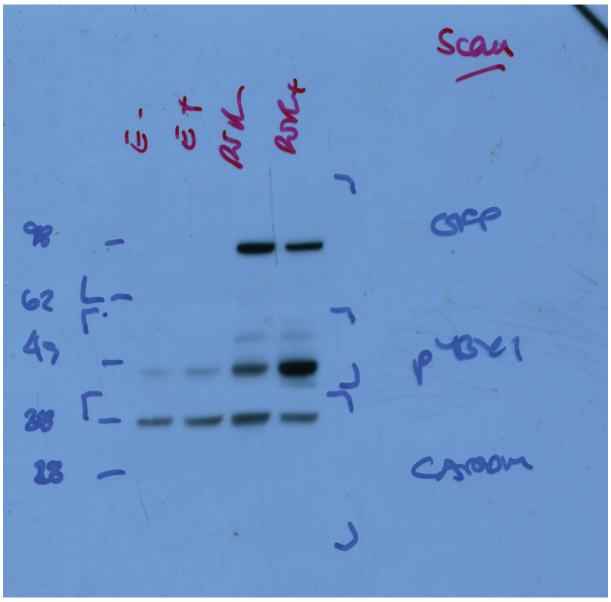

Figure S5f

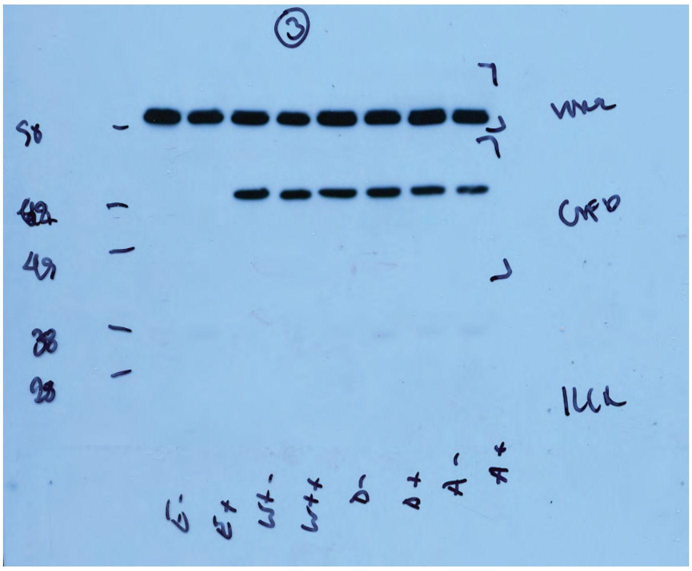

Figure S5g

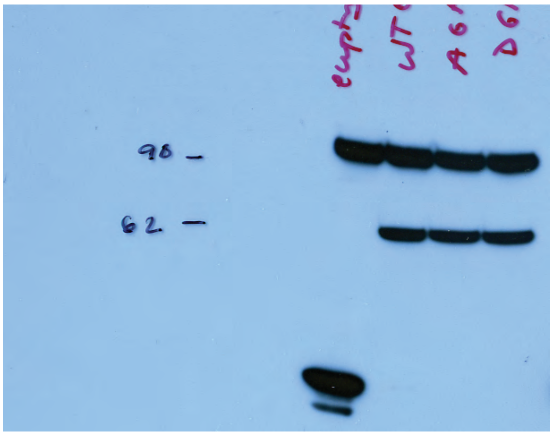

Figure 6a

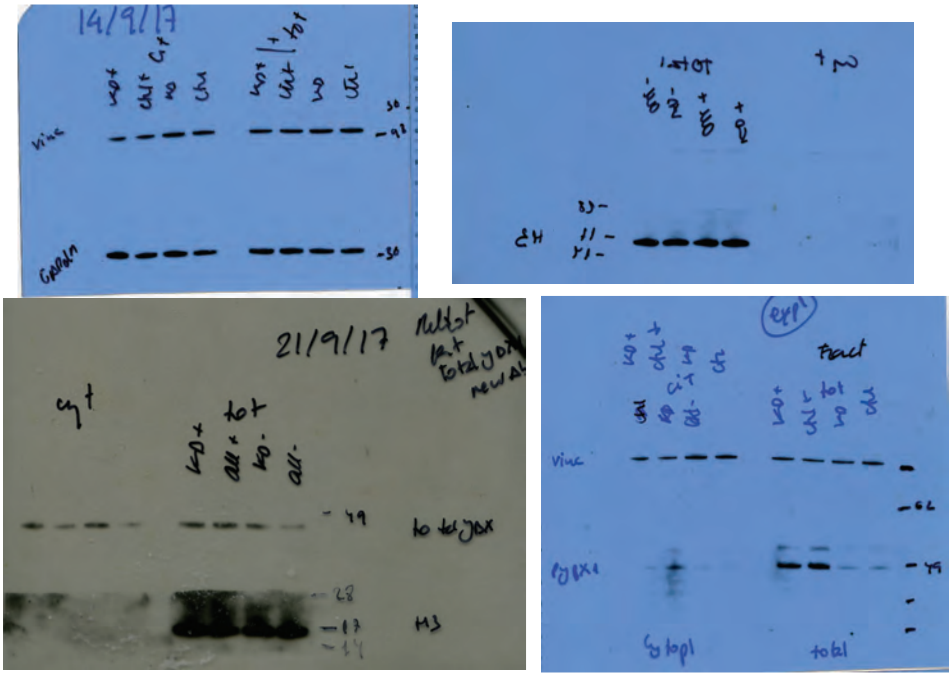

Figure 6e

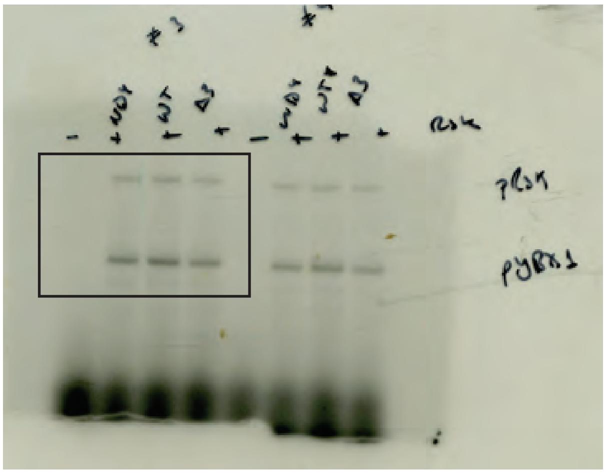

Figure 6f

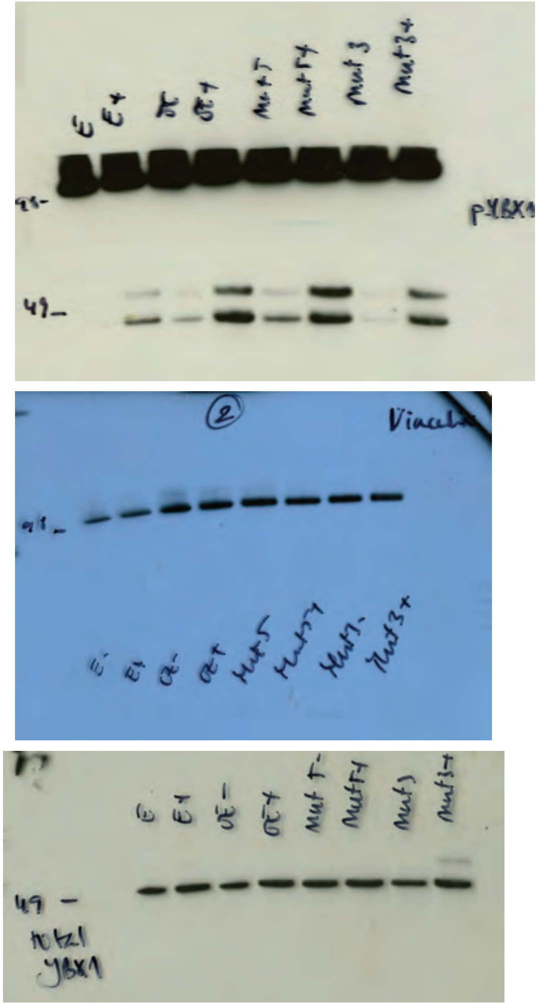

Figure S6b

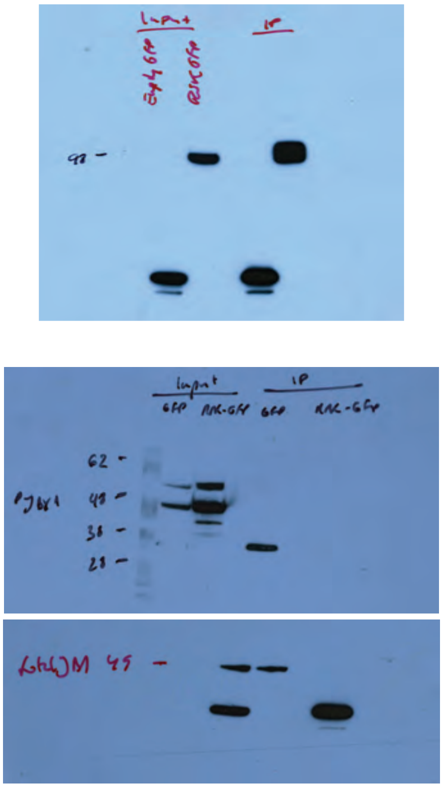

Supplement: Supplementary file 1 — Supplementary Information [file 41467_2021_22746_MOESM1_ESM.pdf]
