# Supplementary material for: The long non-coding RNA MIR31HG regulates the senescence associated secretory phenotype
Source: Nat Commun. 2021 Apr 28;12:2459. doi: 10.1038/s41467-021-22746-4 (PMC8080841; doi:10.1038/s41467-021-22746-4)
Supplement: Supplementary file 3 — Reporting Summary [file 41467_2021_22746_MOESM3_ESM.pdf]

nature research

Corresponding author(s): MARTA MONTES/ANDERS H LUND

Last updated by author(s): Mar 5, 2021

# Reporting Summary

Nature Research wishes to improve the reproducibility of the work that we publish. This form provides structure for consistency and transparency in reporting. For further information on Nature Research policies, see Authors & Referees and the Editorial Policy Checklist .

## Statistics

For all statistical analyses, confirm that the following items are present in the figure legend, table legend, main text, or Methods section.

| n/a | Confirmed | |
|---|---|---|
| ☐ | ☒ | The exact sample size ($n$) for each experimental group/condition, given as a discrete number and unit of measurement |
| ☐ | ☒ | A statement on whether measurements were taken from distinct samples or whether the same sample was measured repeatedly |
| ☐ | ☒ | The statistical test(s) used AND whether they are one- or two-sided<br>*Only common tests should be described solely by name; describe more complex techniques in the Methods section.* |
| ☒ | ☐ | A description of all covariates tested |
| ☐ | ☒ | A description of any assumptions or corrections, such as tests of normality and adjustment for multiple comparisons |
| ☐ | ☒ | A full description of the statistical parameters including central tendency (e.g. means) or other basic estimates (e.g. regression coefficient) AND variation (e.g. standard deviation) or associated estimates of uncertainty (e.g. confidence intervals) |
| ☐ | ☒ | For null hypothesis testing, the test statistic (e.g. $F$, $t$, $r$) with confidence intervals, effect sizes, degrees of freedom and $P$ value noted<br>*Give P values as exact values whenever suitable.* |
| ☒ | ☐ | For Bayesian analysis, information on the choice of priors and Markov chain Monte Carlo settings |
| ☒ | ☐ | For hierarchical and complex designs, identification of the appropriate level for tests and full reporting of outcomes |
| ☒ | ☐ | Estimates of effect sizes (e.g. Cohen's $d$, Pearson's $r$), indicating how they were calculated |

*Our web collection on statistics for biologists contains articles on many of the points above.*

## Software and code

Policy information about availability of computer code

| | |
|---|---|
| Data collection | ZEN pro software (Zeiss) 2011<br>StepOne software v2.3 for Step One Plus real time PCR (Applied Biosystems)<br>Glo-Max multi-detection system with Instict software v3.1.1 (Promega)<br>UCSC Xena platform to download data from TCGA Pan-Cancer |
| Data analysis | ImageJ/Fiji software. version 2.0.0.<br>GraphPad PRISM 8<br>MaxQuant software 72 version 1.5.3.30<br>R environment (https://www.r-project.org) using DEP (v. 1.8.0) bioconductor package for proteomics analysis<br>ggplot2 R package v3.2<br>STAR aligner version 2.5.1a<br>featureCounts version 1.5.1<br>PANTHER79 (http://pantherdb.org/about.jsp) version 14.1<br>Cell Profiler version 3.1.5 |

For manuscripts utilizing custom algorithms or software that are central to the research but not yet described in published literature, software must be made available to editors/reviewers. We strongly encourage code deposition in a community repository (e.g. GitHub). See the Nature Research guidelines for submitting code & software for further information.

# Data

Policy information about availability of data

All manuscripts must include a data availability statement. This statement should provide the following information, where applicable:

- Accession codes, unique identifiers, or web links for publicly available datasets
- A list of figures that have associated raw data
- A description of any restrictions on data availability

The MS data associated with this study has been deposited to PRIDE ProteomeXchange: PXD017475.
RNA-seq data have been deposited in GEO with the accession number: GSE144752

Publicly available data was dowloaded from UCSC Xena platform:
https://xenabrowser.net/datapages/?dataset=tcga_RSEM_gene_fpkm&host=https%3A%2F%2Ftoil.xenahubs.net&removeHub=https%3A%2F%2Fxena.treehouse.gi.ucsc.edu%3A443,
https://xenabrowser.net/datapages/?dataset=mc3.v0.2.8.PUBLIC.nonsilentGene.xena&host=https%3A%2F%2Fpancanatlas.xenahubs.net&removeHub=https%3A%2F%2Fxena.treehouse.gi.ucsc.edu%3A443

The data and reagents that support the findings of this study are available from the corresponding author upon reasonable request.
Source data is available online for Figs. 1–6 and Supplementary Figs. 1–6.

# Field-specific reporting

Please select the one below that is the best fit for your research. If you are not sure, read the appropriate sections before making your selection.

- [x] Life sciences
- [ ] Behavioural & social sciences
- [ ] Ecological, evolutionary & environmental sciences

For a reference copy of the document with all sections, see nature.com/documents/nr-reporting-summary-flat.pdf

# Life sciences study design

All studies must disclose on these points even when the disclosure is negative.

| | |
|---|---|
| Sample size | The majority of the experiments were done in three-four replicates according to standars. No previous study was used to determine the number of samples. |
| Data exclusions | No data was excluded from the analysis |
| Replication | The majority of the experiments were done at least in 3 independent replicates. RNAseq and Mass Spectomerty experiments were validated by qRT-PCR and Immunoblot. |
| Randomization | There was no randomization in the allocation of samples because proper controls were used. All samples (controls and treatments) were treated in the exact same manner for all the experiments. |
| Blinding | Researchers were not blinded during experiments and data analysis because proper controls were used. |

# Reporting for specific materials, systems and methods

We require information from authors about some types of materials, experimental systems and methods used in many studies. Here, indicate whether each material, system or method listed is relevant to your study. If you are not sure if a list item applies to your research, read the appropriate section before selecting a response.

## Materials & experimental systems

| n/a | Involved in the study | |
|---|---|---|
| [ ] | [x] | Antibodies |
| [ ] | [x] | Eukaryotic cell lines |
| [x] | [ ] | Palaeontology |
| [x] | [ ] | Animals and other organisms |
| [x] | [ ] | Human research participants |
| [x] | [ ] | Clinical data |

## Methods

| n/a | Involved in the study | |
|---|---|---|
| [x] | [ ] | ChIP-seq |
| [x] | [ ] | Flow cytometry |
| [x] | [ ] | MRI-based neuroimaging |

## Antibodies

| | |
|---|---|
| Antibodies used | IL6, R&D Systems (AF-206-NA) 1:1000 |

P53 santa cruz (sc-126) 1:2000
p21 BD pharmigen 556431 1:2000
VINCULIN sigma (V 9131) 1:200000
p-RELA cell signaling (3033) 1:1000
RELA santa cruz (sc-8008) 1:1000
CEBPB santa cruz (sc-150) 1:1000
GAPDH santa cruz (25778) 1:2000
IL1A abcam WB 1:1000
IL1A R&D System (MAB200-100) Immunofluorescence 1:100
YBX1 cell signaling (94202S) 1:1000
p-YBX1 (S102) cell signaling (2900) 1:2000
p-RSK cell signaling (11989S) 1:3000
p-AKT cell signaling (4060) 1:1000
AKT cell signaling (9272) 1:1000
LAMIN A1 santa cruz (sc-20680) 1:1000
p-SMAD2 cell signaling (3108T) 1:1000
SMAD2 cell signaling (3103S) 1:1000
IKK cell signaling (2685) 1:1000
IKBa cell signaling 9242 1:1000
p-p70S6K cell signaling (9206) 1:1000
p70S6K cell signaling (9202) 1:1000
p-S6 cell signaling (2211) 1:5000
S6 cell signaling (2217) 1:1000
GFP santa cruz (sc-9996) 1:1000
RSK BD transduction labs (610225) Inmunofluorescence 1:200, WB 1:3000

Validation

Most of the antibodies used for western blot have been used by the Lund lab previously.
Antibodies for IF and PLA experiments were optimized for this study. IL1A R&D System (MAB200-100) 1:100
RSK BD transduction labs (610225) 1:200, YBX1 cell signaling (94202S) 1:100.
Antibody for CHIP was validated by the company (CEBPB abcam ab32358)

## Eukaryotic cell lines

Policy information about cell lines

Cell line source(s)

BJ ER:BRAF and TIG3:BRAF were provided by Professor Kristian Helin, BRIC. MDA-MB-231 cells were provided by Professor Janine Erler (BRIC) originaly from ATCC. BJ wild type cells were originally obtained from ATCC. IMR90 cells were provided by Maite Huarte, CIMA (Spain), originally from ATCC. HEK293T cells were used for virus production and originally obtained from ATCC.

Authentication

BJ-BRAF and BJ cells were authenticated by IMPACT II PCR Profile; CellCheck 9 - human (9 Marker STR Profile and Inter-species Contamination Test) (provided by IDEXX)

Mycoplasma contamination

The cells were routinely tested for mycoplasma infection using Eurofinsgenomics sequencing service and discarded if positive.

Commonly misidentified lines
(See ICLAC register)

No commonly misidentified lines were used
