# Supplementary material for: The long non-coding RNA MIR31HG regulates the senescence associated secretory phenotype
Source: Nat Commun. 2021 Apr 28;12:2459. doi: 10.1038/s41467-021-22746-4 (PMC8080841; doi:10.1038/s41467-021-22746-4)
Supplement: Supplementary file 4 — Description of Additional Supplementary Files [file 41467_2021_22746_MOESM4_ESM.pdf]

# Description of Additional Supplementary Files

**Supplementary Data 1: (a)** (Related to Figure 1C): Normalized reads in control proliferating cells (Control), control senescent cells (Sen) and MIR31HG-depleted senescent cells (Sen siMIR31HG). **(b)** (Related to Figure 1C): Outcome from DeSeq2. **(c)** (Related to Figure 1D): Outcome from DeSeq2 comparing control senescent cells (Sen) and MIR31HG-depleted senescent cells (Sen siMIR31HG)**. (d)** (Related to Figure 1D): Normalized reads in control senescent cells (Sen) and MIR31HG-depleted senescent cells (Sen siMIR31HG) for a subset of SASP genes.

See materials and methods for details on sample preparation and data processing.

**Supplementary Data 2: (a)** (Related to Figure 1F): Proteins identified in secretome of control proliferating cells (Control), control senescent cells (Sen) and MIR31HG-depleted senescent cells (Sen siMIR31HG). Table is sorted by decreasing fold change between MIR31HG knockdown and control siRNA treatment upon senescence ('Sen siMIR31HG_vs_Sen_ratio'). Non-senescent control is included for comparison. Unprocessed data can be found in Suppl. Table 2B. **(b)** (Related to Figure 1F): Unprocessed MaxQuant results file corresponding to Suppl. Table 2A; parameters description can be found in MaxQuant documentation (Cox, J. and Mann, M. Nat Biotechnol, 2008, 26). **(c)** (Related to Figure 4B):  Proteins detected in MIR31HG (MIR31HG) and Luciferase (CTRL) RNA pulldown experiments using antisense oligonucleotides for enrichment. Table is sorted by decreasing fold enrichment of MIR31HG over Luciferase control. Unprocessed data can be found in Suppl. Table 2D. For information about data processing see Materials and Methods. Supplementary **(d)** (Related to Figure 4B):  Unprocessed MaxQuant results file corresponding to Suppl. Table 2C; parameters description can be found in MaxQuant documentation (Cox, J. and Mann, M. Nat Biotechnol, 2008, 26).

**Supplementary Data 3: (a)** (Related to Figure 4C)Normalized reads in control proliferating cells (Control), control senescent cells (Sen) and MIR31HG-depleted senescent cells (Sen siMIR31HG). **(b)** (Related to Figure 4C): Normalized reads in control senescent cells (Sen) and MIR31HG-depleted senescent cells (Sen siMIR31HG) for a subset of SASP genes
